# What do we know about patient-provider interactions in sub-Saharan Africa? a scoping review

**DOI:** 10.11604/pamj.2020.37.88.24009

**Published:** 2020-09-25

**Authors:** Bienvenu Salim Camara, Loubna Belaid, Hawa Manet, Delphin Kolie, Etienne Guillard, Théophile Bigirimana, Alexandre Delamou

**Affiliations:** 1 Centre National de Formation et de Recherche en Santé Rurale de Maferinyah, Forécariah, Guinea,; 2 Family Medicine, McGill University, Montreal, Quebec, Canada,; 3 Solthis, Paris, France,; 4 Solthis Guinée, Conakry, Guinée,; 5 Department of Public Health, Gamal Abdel Nasser University of Conakry, Conakry, Guinea

**Keywords:** Patient-centred care, patient-provider interaction, sub-Saharan Africa, scoping review

## Abstract

**Introduction:**

patient-centred care has become a rallying call for improving quality and access to care in countries where health system responsiveness and satisfaction with health services remain low. Understanding patient-provider interactions is important to guide implementation of an effective patient-centred care approach in sub-Saharan Africa. This review aims to overcome this knowledge gap by synthesizing the evidence on patient-provider interactions in sub-Saharan Africa.

**Methods:**

we conducted a scoping review using Arksey and O´Malley´s framework. We searched in eight databases and the grey literature. We conducted a thematic analysis using an inductive approach to assess the studies.

**Results:**

of the 80 references identified through database searching, nine met the inclusion criteria. Poor communication and several types of mistreatment (service denial, oppressive language, harsh words and rough examination) characterize patient-provider interactions in sub-Saharan Africa. Nevertheless, some health providers offer support to patients who cannot afford their medical expenses, cost of transportation, food or other necessities. Maintaining confidentiality depends on the context of care. Some patients blamed health providers for consulting with the door open or carrying out concomitant activities in the consultation room. However, in the context of HIV care provision, nurses emphasized the importance of keeping their patients´ HIV status confidential.

**Conclusion:**

this review advocates for more implementation studies on patient-provider interactions in sub-Saharan Africa so as to inform policies and practices for patient-centred health systems. Decision-makers should prioritize training, mentorship and regular supportive supervision of health providers to provide patient-centred care. Patients should be empowered in care processes.

## Introduction

Patient-centred care has gained the attention of policymakers and health managers over the last decade, especially in low- and middle-income countries [1-4]. It has been identified as a key component of quality of care [3], and has been recognized by the World Health Organization (WHO) as a core competency of health workers to transform health care [5]. Patient-centred care has therefore become a rallying call for improving quality and access to care in countries where health system responsiveness and satisfaction with health services remain low [4]. Patient-centred care is defined as “providing care that is respectful of, and responsive to, individual patient preferences, needs and values, and ensuring that patient values guide all clinical decisions” [6]. This implies that patient-centred care provision and evaluation rests on understanding patient-provider interactions (PPI). In sub-Saharan Africa, PPI in different contexts of care have shown health providers disregarding patients´ views or infringing their dignity in the process of care. For instance, in Rwanda, patients seeking care in a primary healthcare setting reported that health providers did not listen to them and behaved in an unfriendly manner [7]. In South Africa, health providers were reported as lacking confidence in women´s ability to make appropriate choices at the time of birth and consequently prioritized biomedical care rather than patient-centred care during delivery [8]. Patients in different countries have also reported being abused, scolded and receiving insults from health providers during antenatal care, labour, or when they came to collect anti-retroviral therapies (ART) [8-12]. However, some health providers have been reported by patients as being kind [13], caring [14, 15], helpful [13, 14], compassionate [14, 15], or as providing enough information to the patient about the process of care [13, 16].

Findings on PPI are inconsistent across countries in sub-Saharan Africa, probably due to the lack of interest in the issue, the complexity of care provision from one type of care to another, but also to the different environments in which the care is provided. Understanding PPI in these different contexts plays an important role in guiding implementation of an effective patient-centred care approach in sub-Saharan Africa. In high-income countries, the key ingredients for implementing patient-centred care have been described. One key illustration is the dimensions of patient-centredness conceived by Mead and Bower [17]. Few studies describing PPI in different African countries have attempted to identify the dimensions of patient-centred care [7, 8, 13]. Since the patient-provider relationship is a core component of patient-centered care, there is, therefore, a need to improve understanding and provide information on how to design patient-centred care in sub-Saharan Africa. This review aims to close this knowledge gap by synthesizing the evidence on PPI in sub-Saharan Africa.

## Methods

We followed the methodological frameworks from Arksey and Levac to conduct the scoping review [18]. This is an appropriate method for examining complex health phenomena such as patient and health provider interactions and does not exclude studies based on methodological criteria. With the support of a medical librarian, we conducted research in several electronic databases (EMBASE (OVID interface, 1974-2015) CINAHL, Ovid Medline (OVID interface, 1946-2015) , Medline (PubMed interface, 1975-2015), ERIC, Psyc INFO, Social Work, soc Index and Reviews, Social Sciences Abstracts), and in the grey literature (Google Scholar, Social Care Online) from 1990 to 2018 (April, 2018).

The concept of “patient centered care” emerged in the 1990s. We defined a search strategy for each database. Furthermore, we scanned the references of the selected references to identify additional studies. The search languages were restricted to French and English.

Two reviewers made an independent assessment of all the potentially eligible studies for inclusion. Discrepancies between the two reviewers were resolved by consensus with the opinion of a third member of the research team. The full search strategy, including search fields, retrievals and inclusion/exclusion criteria is included.

We conducted a thematic analysis using an inductive approach. We adopted an intermediate approach to assessing the quality of the studies, recommended for reviews combining quantitative with qualitative studies [19]. We used the PRISMA checklist for scoping review (PRISMA-ScR).

### Funding

Solthis (Solidarité Thérapeutique et initiatives pour la Santé) and the Agence Française de Développement (ADF) funded this study.

## Results

### Description of the studies included

**Figure 1:**
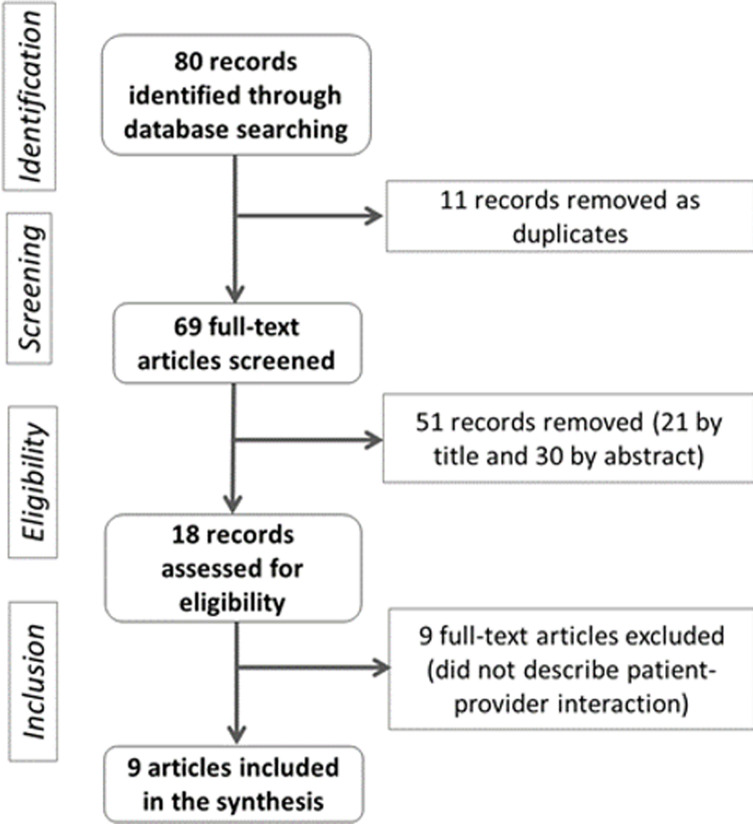
flow chart of the studies´ selection process

We included nine studies in this scoping review (Figure 1). The studies included were published between 2013 and 2018 in ten sub-Saharan African countries: Rwanda [7], Mali [14], South Africa [8, 20], Ethiopia [16], Tanzania [13, 15], Malawi [11], and one a multiple settings study (Kenya, Malawi, Namibia, Rwanda, Senegal, Tanzania, Uganda) [21] (Table 1). Eight studies were conducted in government health facilities and one in both government and NGO health facilities. Six studies were qualitative, two used a mixed-method design [11, 16], and one was a quantitative one [21]. Five studies explored the interaction between health providers and patients in HIV care [11, 13-16], three in maternal care [8, 11, 13] one in family planning services [20], one in child health care [21] and one in adult primary health care [7].

**Table 1:** characteristics of the studies included in the synthesis

Author and year	Location and setting	Study objectives	Study design	Study population and sample size
Cubaka *et al*., 2018 (7)	Rwanda (urban and rural health centres)	To gain more insights into patients´ perceptions of their interactions with nurses in primary care settings in Rwanda.	Qualitative study	15 patients aged above 21 years old
Hurley *et al*., 2018 (14)	Mali (urban government health centres, NGO clinics, teaching hospitals)	1) To describe the features of provider-patient communication that patients value in their care experience. 2)To explore how provider-patient communication may alter the trajectory of disengagement by examining how these features intersected with experiences of barriers to engagement and reengagement	Qualitative study	69 HIV patients (engaged for ART and disengaged for ART) and 17 HIV care providers
Lambert *et al*., 2017 (8)	South Africa (urban and rural public health facilities)	To explore experiences of care during labour and birth from the perspectives of both the healthcare provider and women receiving care, to inform recommendations for how the quality of care can be improved and monitored, and, to identify the main aspects of care that are important to women.	Qualitative study	49 women who have given birth in the preceding 12 weeks, 33 healthcare providers working in the labour ward, 10 managers and policy makers
Larson *et al*., 2017 (21)	Kenya, Malawi, Namibia, Rwanda, Senegal, Tanzania, Uganda (Hospitals and health centres)	To determine the extent of provider communication, predictors of good communication and the association between provider communication and patient outcomes, such as patient satisfaction, in seven sub- Saharan African countries.	Cross-sectional, multi-country study	16,352 caregivers visiting the facility for their sick children.
Tiruneh *et al*., 2016 (16)	Ethiopia (HIV clinic, Teaching Hospital of Addis Ababa)	1) To assess whether or not patients take their medicine (adherence to dose) and the extent to which they follow the prescribed regimen (including adherence to dosing schedule). 2)To understand the sociocultural context within which HIV patients understand and relate to their ART adherence requirements, especially regarding regular dose timing.	Mixed methods study	105 HIV patients receiving ART
Gourlay *et al*., 2014 (13)	Tanzania (rural government dispensaries and health centre in Kisesa)	To explore the nature of patient-provider interactions within PMTCT service provision in rural Tanzania, and ways in which these interactions influence the uptake of PMTCT services, with the aim of providing recommendations for optimising patient-provider relations and PMTCT uptake.	Qualitative study	Mothers and fathers, HIV positive and HIV negative patients, health providers
Marlow *et al*., 2014 (20)	South Africa (urban government health clinic)	To better understand health facility factors influencing women´s post-partum contraceptive use.	Qualitative study	14 family planning clients and five nurses who promoted family planning post-partum
Iroezi *et al*., 2013 (11)	Malawi (rural hospital and referring clinics	To address the barriers and facilitators to HIV care for pregnant and postpartum women in Malawi	Mixed methods study	22 HIV-infected pregnant and postpartum women
Vaga *et al*., 2013 (15)	Tanzania (rural clinic and within communities)	To explore how nursing care emerges, i.e. how care is experienced and expressed in nurses´ everyday work among HIV positive women enrolled in a PMTCT programme.	Qualitative ethnographic study	HIV-infected women and nurses

### Patient-provider interactions (PPI)

We synthesized PPI described in the selected studies under seven themes including: communication, understanding, respect and compassion for the patient, stigmatization, decision-making, and trust.

### Communication

Communication has been reported as an important dimension of PPI. The studies addressing this dimension explored it from the patient perspective. Communication referred to welcoming patients, listening to them and giving them explanations concerning their care. The two studies that mentioned the reception of patients reported mainly on patients´ perceptions of the manner in which they were seen by health providers [7, 14]. Patients emphasized that it was important that health providers receive them in an acceptable way [7] and felt that a warm welcome was the starting point for establishing a positive rapport [14]. They considered a warm welcome to be one where health providers smiled at the beginning of the consultation, showed that they were happy to see the patient, demonstrated an interest in the patient´s well-being, asked about family, and offered extended personal greetings and blessings according to local customs [14]. In Mali, in a context of HIV care, patients reported that health providers used humour, such as traditional “joking cousins” (teasing aligned with rival family surnames). They said that such health provider attitudes made them feel welcome and comfortable.

*“•••The good doctors get close to you, they greet you well, and that really makes people happy.”* [14].

However, some of the patients felt distanced from health providers who had a “grimace on their face”, did not welcome them warmly or used French or medical jargon whereas the patients spoke mainly the local language (Bambara) [14].

*“If the doctors say something in French, that means it is not something good. It is all the talking that [the doctors] do together•••”* [14].

Two studies addressed health providers listening to their patients [7, 14]. In the study conducted in Rwanda [7], several patients felt they were being rushed during the consultations. *“There are health care providers who even finish prescribing medications for you before you finish talking and just give you the paper and you go.”* [7]. In Mali, patients felt that health providers who took the time to listen to them not only helped to solve their problems but valued them as well. They therefore recommended that health providers should find the time outside consultation times to listen to patients with complex problems [14].

*“If that person comes and the doctor knows that they have a lot of problems, [the doctor] must listen to them. The doctor can say, “So-and-so, I am listening to you. But since there are some people waiting, I am going to give you an appointment for this day.” [•••] Or: “You can sit and wait a little. If the people decrease, I will take you; because it is pleasing to me to listen to you.”* [14] Most of the studies explored the explanations given to patients. Regarding health providers´ explanations to patients, a cross-sectional study which assessed health providers´ communication tasks with caregivers seeking care for their children in seven African countries, it reported that 54% of caregivers said they were informed of the child´s diagnosis and only 10% received counselling on feeding or providing liquids to the child [14]. Qualitative studies in a context of general [7, 20] as well as HIV care [13, 16] reported that patients were denied an explanation about their physical health, paramedical examination, diagnosis and treatment. A 32-year-old female HIV patient seeking family planning in South Africa reported [20]: [The nurse] asked if I chose pills or the injection, I said the injection for 3-months and she wrote it down, and I got the injection. They don´t explain a lot of things; you just say the injection that you want or the pills that you want, that´s all.” [20].

In Ethiopia, Tiruneh *et al*. observed that conversations between providers and patients in HIV counselling sessions were short, consisted of instructions and did not enable patients to ask questions or express their concerns. *“In a number of counselling sessions with HIV patients, conversations between patients and healthcare workers regarding medication adherence were short and too prescriptive to reasonably address any concern from the patients´ perspectives. The provider speaks and the patient listens; the latter do not seem to sense their entitlement to ask questions, much less to delve into practicalities [field note],”* [16]. Some articles showed that patients´ willingness to engage in conversation with health providers was hindered by the latter´s attitudes that were perceived as detached or arrogant [7, 13-15]. A patient in Mali reported: *“If you behave like you are more important than them, even if something is bothering them, they will not tell you. [•••] If they are scared of the doctors, they don´t tell.”* [14].

Patients also felt reluctant to talk about their problems when health providers seemed rushed, focused on their phone, computer or paperwork, or when there were several people in the consultation room, e.g. trainees or interns [8].

### Understanding the patient

Few studies discussed this aspect of PPI [8, 14, 20]. Understanding patients´ requests was a theme addressed by Lambert *et al* in South Africa [8]. They found that health providers were blamed by patients for denying their requests in the process of care. Many women reported being refused visits to the delivery ward of a close relative, such as the mother or partner. *“During that time that was terrifying for me, they must allow at least the boyfriend or the husband or someone. That was the important thing to me, not be alone at that time. When my boyfriend couldn´t come in, they didn´t respect my needs.”* [8].

Another theme discussed was understanding patients in the event of a missed appointment. In facilities offering ART services in Bamako (Mali), health providers were valued by the patients as they understood the patients´ personal difficulties regarding adherence to the care process. For instance, they helped with arranging for patients to get medication by other means when the patient was travelling or for medication to be taken to a patient who was unable to leave home [14].

However, re-engagement of patients who defaulted from medication was sometimes accompanied by scolding from health providers, or referrals to a psychosocial counsellor or ART education sessions. These practices were perceived by patients as a shortcoming on the part of the health providers, a punishment for noncompliance [14]. Similarly, in South Africa, a family planning client felt that nurses were not giving the patients optimal care because the latter had arrived at the clinic later than scheduled in the day [20]: “*The nurses asked me why I came in the afternoon. I told them that I forgot that it was the 16^th^and that was why I didn´t wake up early in the morning to come to the clinic. When I checked my card in the afternoon and saw that it was my date, I rushed and came late. [Because it was so late] the nurses didn´t have any time. I don´t want to lie to you; they didn´t have any time*. [18].

### Respecting patients

Respect for a patient was defined as any attitude on the part of the provider that was likely to maintain the patient´s dignity. The four articles that discussed this aspect used qualitative methods [7, 8, 11, 13]. We categorized the themes emerging from these studies into two themes: mistreatment of the patient and distraction on the part of the health provider. All these studies mentioned that patients testified to being mistreated by health providers during care provision. It included oppressive or judgmental language [7], harsh words when patients came to collect their medications, including ARTs [11, 13], scolding and rough examination during antenatal care visits, verbal and physical abuse during labour [8, 11]. An HIV positive woman seeking ART in Malawi reported: *“•••When you come to get medication here, before he gives you the medication, he will rain insults at you first•••”* [11].

One study mentioned that, despite recognizing the behaviour they were blamed for, health providers believed that it was an essential part of care in labour to speak firmly or shout. They also justified such behaviour as necessary in the labour process because of pressure resulting from being busy and short-staffed. *“You were trying to save the baby. You were not necessarily shouting at her but just trying to get her to do the right thing”* [8].

However, in this study, a woman indicated receiving respect and help from health providers when she was in labour [8]: *“•••They helped me to the bed and then they dressed me and “Please can you just open the legs, nicely, and hold it and just relax. The baby is on its way. They were so nice•••”* [8].

The study on adult health care in a primary care setting in Rwanda discussed health provider distraction in the process of care [7]. In this study, patients felt that health providers´ distraction was a serious problem. They accused the latter of being distracted during the consultation, talking on their phones, talking to colleagues or writing. *“He just looks down onto his paper and keeps writing as if not realizing my presence; but it would be better if he would turn and look at me while he asks me questions and then turn away when he wants to write.”* [7].

### Compassion for the patient

We considered compassion for the patient as any personal support from the health provider to the patient in the process of care. Several studies discussed this aspect [11, 13-15]. Patients and health providers gave examples of health providers offering support to those patients who could not afford their medical expenses, transportation, food or other necessities [13, 14]. Other supports improved responsiveness to patients´ needs [14, 15], including : providing medication to patients based on their personal schedules, mediating conflicts with their families or community members, linking patients with support groups and sometimes helping to transfer them to other facilities. In a prevention of mother to child transmission of HIV (PMTCT) programme in rural Tanzania, Gourlay *et al*. also observed that above and beyond planned home visits, nurses frequently made short courtesy visits to greet and check on HIV patients enrolled in their programme as they passed their houses on their way to home visits. The observers reported that *“we can´t pass without saying hello”*, and *“I just want to check on her”* were commonly heard [13]. Gourlay *et al*. also testified that health providers had comforting talks with HIV patients who were feeling anxious about the difficulties of coping with life and their babies. In HIV care in Mali, patients also acknowledged that health providers helped to calm their mind in times of anxiety, uncertainty or emotional distress, notably when they were first diagnosed with HIV [14] *“They joked with me about my baby, and I became at ease. I thought I had a big problem, but they really calmed my mind. You see? Sometimes, you even forget that they are doctors.”* [14]. Patients found such health providers´ attitudes not only emotionally reassuring but an essential and direct component of the healing process [14].

### Patient stigmatization

One study in Tanzania [13] reported HIV patients suffering stigma from health providers during antenatal care and labour. This was expressed by verbal insults, by not receiving services or being told by nurses to wash their own clothes after delivery. *“That nurse•••sometimes she would tell you•••“you are suffering from AIDS, you will also give birth to a baby with diseases, you will suffer.””*. [13]. *“•••[health providers tell you that] you are supposed to wash them [delivery clothes] yourselves•••so that you may not infect the person who is attending you*”. [13].

### Decision-making

Few studies - one from Rwanda and two from Tanzania - discussed decision-making in the process of care [7, 13, 15]. These studies showed that both patients and health providers perceived that decision-taking in the process of care should be done by the health provider. In rural Tanzania, Gourlay *et al*. observed that the use of terminologies such as “experts”, “specialists” and “experienced” by patients and the use of expressions such as “ordered” or “violating the conditions” by health providers was common [13]. In South Africa, Lambert *et al*. [8] did not discuss directly the decision-making process in their study exploring the experience of care during labour and birth. However, they interpreted that health providers, managers and health policy makers seemed to hold the common view that women in labour were not able to make the right decisions and should be told what to do.

*“Keep on reminding them what they have to do, even when they have had a baby. They make mistakes.” Nurse-Midwife, FGD* [8]. For their part, patients regarded health providers as the people who possessed the answers to their problems. They were the individuals who knew better than they did, who were there to give them instructions [7, 13, 15]. A male patient seeking care at a health centre in Rwanda said: *“•••I have to be below her/him as we are not at the same position. Anyway, it is the reason I come to a health facility so that the clinician - who is above me, who has knowledge, who has learnt/studied something - gives me a piece of advice on how I should do/behave”* [7]. The studies also reported that making decisions for their health was poorly understood and perceived by patients as compromising. Where patients are concerned, this may result in deviating from treatment guidelines [7] or being denied health services [13], as mentioned by a male patient in Rwanda and a female HIV patient in rural Tanzania: *“We would probably make bad decisions for ourselves. We could say for example they would give me two pills when they should give me four pills; and that would not be good for me.”* [7]. *“You won´t be provided service if you are not tested [for HIV]•••you can´t refuse, you must be tested.”* [13].

### Trust

We referred to this concept as the belief patients and health providers have in each other in terms of care. The studies that addressed this aspect discussed health providers´ confidentiality [7, 13], competency [13], morale related to their own benefit [13], and their mistrust in patients [7, 8]. Where confidentiality is concerned, patients accused health providers of consulting with the door left open or of carrying out concomitant activities in the consultation room, and therefore of not respecting their privacy. This resulted in some of them preferring to avoid disclosing all their problems to health providers even though they felt very sick [7].

*“••• Sometimes, you become silent as you feel there are things you cannot tell her/him while you went there being very sick”* [7]. However, in a context of HIV care provision in rural Tanzania, nurses emphasized keeping their patients´ HIV status confidential. One commented: *“I don´t think there is a worker giving away secrets”*, and another mentioned the potential implications for patient retention: *“when you give away that secret, that patient will not come again”*. In addition, nurses mentioned that some HIV women were anxious when they were referred to other health providers (e.g. due to a lack of ARVs or HIV test kits), after having established a relationship of trust with their first health provider [13].

*“If she starts being given the service by you, it will be you only•••, she doesn´t want another worker to know that thing [her status]•••”* [13]. Health providers´ competency was addressed briefly in the study in rural Tanzania, reporting that providers were subject to mistrust because of their incompetency, such as apparent loss of test results or scepticism regarding HIV test results [13]. However, in Rwanda, Cubaka *et al* [7] found that patients, mainly those with limited literacy, had a kind of blind and naïve trust or faith in providers. A female patient reported: *“When he tells you something, he has a reason why he tells you so [•••] the health care provider cannot prescribe something bad for you.”* [7].

## Discussion

To our knowledge, this scoping review is the first to describe the interactions between patient and provider (IPP) in diverse contexts of care in sub-Saharan Africa. Its findings highlight the complexity of PPI that needs to be understood in the light of the impact on health policies and practices in an era that promotes patient-centred care. Three main reasons might explain why health providers interact poorly with patients. First, the training undergone by health providers is exclusively biomedical i.e. diagnosis and provision of drugs or surgical services, with insufficient focus on learning how to communicate with patients, how to take into account the other dimensions of the patient, such as the psychosocial well-being and cultural and socio-economic aspects [22, 23]. Second, the social status given by patients to biomedical health providers, especially physicians, makes them feel superior to the patient when providing care [8, 13]. Furthermore, this social relationship has been normalized by patients who believe that only the health provider knows best about their health, who should decide and instruct them about their care [7, 13, 15]. In Africa, the power imbalance might have increased because of the disempowerment of the patient created by the low educational level and/or low social and economic status [24, 25]. This power given to health providers might explain the poor communication that is apparent between the patient and the health provider. Third, it is important to emphasize the inappropriateness of the health systems in the provision of patient-centred care in sub-Saharan Africa. These health systems are characterized by a shortage of skilled staff, resulting in an excessive workload for existing staff, and thus less time is dedicated to consultations with patients [20, 26]. Furthermore there is low staff motivation [13], a lack of staff training in communication [21, 27], poor supervision [21], poor health provider accountability in the provision of care (e.g. no sanctions in the event of patient abuse by the health provider) [28] and poor infrastructure to ensure patient confidentiality and protection of privacy [7].

However, this scoping review reports positive behaviour on the part of some health providers although it is not clear why some of them continue to have positive interactions with patients, despite the many obstacles mentioned above. Indeed, a limited number of studies has reported the positive aspects of health providers´ attitudes compared to the literature, which puts great emphasis on poor health provider attitudes. This publication bias is likely to hamper understanding of PPI in sub-Saharan Africa. It is, therefore, important to close this research gap by endeavouring to understand what works well and which methods are used in the provision of care to patients. Health providers were reportedly more compassionate when providing HIV care [13, 14] than in the provision of childbirth care [8, 13, 29]. One explanation could be the particular training and supervision of health providers working in HIV programmes (where emphasis is on the psychosocial dimensions of the disease) [30], compared to childbirth. Nonetheless, there is a need to improve understanding of the reasons for rude behaviour on the part of childbirth care providers in order to render maternal health care more patient-centred in sub-Saharan Africa [10].

Moreover, it is important to note that many publications have shown how vulnerable the patients are, disempowered in the process of care, especially in sub-Saharan Africa [11, 13, 20]. More research is needed on how to take the preferences of patients into account and engage them in decisions related to their care. Closing these research gaps is the key to guiding policies towards patient-centred care. Based on the findings of this review, there are three core dimensions that need to be taken into account to implement patient-centred care in sub-Saharan Africa. The first dimension is “the patient as a person´. This dimension expects the health provider to welcome patients, listen to them, share all the necessary information they might need, seek their consent and treat them with respect. The second dimension, which is the provider as a person, calls for the demystification of health providers. Health providers should defer their identity as experts and try to establish a link with the patients in line with their cultural norms and spoken language, understanding their personal problems, and expressing friendliness and compassion. The third dimension, the provider as confidant, recommends that the health provider build and maintain patients´ trust and encourage them to engage in conversation. However, to implement these dimensions, health systems should give priority to training that focuses on patient-centred care, along with mentorship.

The present scoping review has some limitations. The geographical distribution of selected studies (sub-Saharan Africa) could affect the external validity of the findings in other low-income countries. The diversity of contexts of care (HIV, primary care, maternal and reproductive health) and perspectives (nurses, midwives, patients) of the studies show the diversity of PPI but also the challenges involved in drawing general conclusions.

## Conclusion

This scoping review shows that poor communication and several types of mistreatment (service denial, oppressive language, harsh words, stigma and rough examination) characterize PPI in sub-Saharan Africa. However, some health providers do offer support to patients who cannot afford their medical expenses, transportation, food or other necessities. Maintaining confidentiality depends on the context of care. More research is needed so as to understand PPI in different contexts of care with a view to improving the design of patient-centred care interventions. Furthermore, decision-makers should prioritize training, mentorship and regular supportive supervision of health providers to provide patient-centred care. Finally, patients should be empowered in the process of care. Regular audits of PPI should be conducted to assess health providers´ accountability.

### What is known about this topic

- Patient-centred care has been identified as a key component of quality of care;
- Perception of maternal health services users about quality of care vary across contexts in sub-Saharan Africa;
- Patient-provider interaction has been described in high-income countries.

### What this study adds

- Patient-provider interaction in sub-Saharan Africa is characterized by poor communication, service denial, oppressive language, harsh words, and rough examination;
- Maintaining confidentiality depends on the context of care.
